# Genetic Diversity of Common Bean (*Phaseolus vulgaris* L.) Landraces Based on Morphological Traits and Molecular Markers

**DOI:** 10.3390/plants13182584

**Published:** 2024-09-15

**Authors:** Evaldo de Paula, Rafael Nunes de Almeida, Talles de Oliveira Santos, José Dias de Souza Neto, Elaine Manelli Riva-Souza, Sheila Cristina Prucoli Posse, Maurício Novaes Souza, Aparecida de Fátima Madella de Oliveira, Alexandre Cristiano Santos Júnior, Jardel Oliveira Santos, Samy Pimenta, Cintia dos Santos Bento, Monique Moreira Moulin

**Affiliations:** 1 Instituto Capixaba de Pesquisa, Assistência Técnica e Extensão Rural (INCAPER), 60 Kurt Lwein Av., Domingos Martins 29273-700, ES, Brazil; evaldodepaula1969@gmail.com (E.d.P.); elaine.riva@gmail.com (E.M.R.-S.); cristinaposse4@gmail.com (S.C.P.P.); 2 Laboratory of Agricultural Engineering, Universidade Estadual do Norte Fluminense Darcy Ribeiro, 2000 Alberto Lamego Av., Campos dos Goytacazes 28013-602, RJ, Brazil; almeida.rna94@gmail.com; 3 Laboratory of Genetics and Plant Breeding, Universidade Estadual do Norte Fluminense Darcy Ribeiro, 2000 Alberto Lamego Av., Campos dos Goytacazes 28013-602, RJ, Brazil; 4 Instituto Federal de Educação, Ciência e Tecnologia do Espírito Santo—Campus de Alegre, Rodovia ES-482 (Cachoeiro-Alegre, Km 72), Alegre 29500-000, ES, Brazil; jdiassneto@gmail.com (J.D.d.S.N.); mauricio.souza@ifes.edu.br (M.N.S.); amadella@ifes.edu.br (A.d.F.M.d.O.); alexandre.cristiano@ifes.edu.br (A.C.S.J.); 5 Department of Biology, Universidade Federal do Piauí, 1401-1519 Dirce Oliveira St., Teresina 64048-550, PI, Brazil; jardel_santos@ufpi.edu.br; 6 Department of Agriculture, Universidade Estadual de Montes Claros (UNIMONTES), 2630 Reinaldo Viana St., Janaúba 39440-000, MG, Brazil; samy.pimenta@unimontes.br; 7 Laboratory of Plant Breeding, Universidade Federal do Espírito Santo (UFES), Alto Universitário, Alegre 29500-000, ES, Brazil; cintia.bento@ufes.br

**Keywords:** Germplasm bank, morphological characterization, molecular characterization, ISSR, genetic variability, plant genetic resources

## Abstract

The objective of this study was to evaluate the genetic diversity among traditional common bean accessions through morphological descriptors and molecular markers. Sixty-seven common bean accessions from the Germplasm bank of the Instituto Federal of Espírito Santo—Campus de Alegre were evaluated. For this, 25 specific morphological descriptors were used, namely 12 quantitative and 13 qualitative ones. A diversity analysis based on morphological descriptors was carried out using the Gower algorithm. For molecular characterization, 23 ISSR primers were used to estimate dissimilarity using the Jaccard Index. Based on the dendrograms obtained by the UPGMA method, for morphological and molecular characterization, high genetic variability was observed between the common bean genotypes studied, evidenced by cophenetic correlation values in the order of 0.99, indicating an accurate representation of the dissimilarity matrix by the UPGMA clustering. In the morphological characterization, high phenotypic diversity was observed between the accessions, with grains of different shapes, colors, and sizes, and the accessions were grouped into nine distinct groups. Molecular characterization was efficient in separating the genotypes in the Andean and Mesoamerican groups, with the 23 ISSR primers studied generating an average of 6.35 polymorphic bands. The work identified divergent accessions that can serve different market niches, which can be indicated as parents to form breeding programs in order to obtain progenies with high genetic variability.

## 1. Introduction

Beans play an essential role in maintaining food security and combating malnutrition, especially in regions severely affected by poverty, as they contain an average of 22% protein [1,2]. It is estimated that the common bean is the primary source of nutrients for over 300 million people worldwide who include beans in their daily diets [3]. The consumption of beans presents several health benefits, such as antioxidant, anti-inflammatory, anticarcinogenic, antihyperglycemic, and even antihypertensive properties [4]. Despite beans’ significant importance for promoting human health and preventing a substantial number of diseases, the production and consumption of the common bean have been experiencing a sharp decline year after year. This decline has created a concerning scenario where many genotypes are disappearing [5,6,7].

In Brazil, the common bean is predominantly cultivated by small farmers. It is estimated that 60% of the national production of the common bean comes from family farming [8]. The global production of the common bean is around 27.5 million tons, with Brazil ranking third in the world, producing a total of 3.2 million tons, behind Myanmar and India, respectively [3]. However, both consumption and planted areas have been declining over the years; in the 1981/1982 harvest season, it was 6155 hectares, whereas in 2022/23, it was only 2699 hectares [9]. This highlights an ongoing process of genetic erosion in the *Phaseolus* genus, especially in the southeastern region of the country, due to agricultural modernization, the replacement of local varieties with commercial cultivars, disease occurrence, the abandonment of agricultural activities, labor shortages, and low bean prices, among other factors [5].

The common bean (*Phaseolus vulgaris* L.) is one of the most significant legumes used in human nutrition, being of great economic, nutritional, and cultural importance throughout Brazil [10]. The genus *Phaseolus* includes a considerable number of species, but only five species are domesticated: the common bean *(P*. *vulgaris* L.), runner bean (*P. coccineus* L.), year bean (*P*. *polyanthus* Greenman), lima bean (*P. lunatus* L.), and tepary bean (*P. acutifolius* A. Gray) [11]. The cultivation of common bean originates from the American continent, with two gene pools (centers of origin): one in the Andean region and the other in the Mesoamerican region. Brazil is an important secondary center of diversity for this crop, with a vast variability of germplasm, exhibiting different shapes, colors, and sizes [11]. In Brazilian territory, there are beans of both Andean and Mesoamerican origin, with the latter predominating (it is estimated that 80% of bean varieties in the country are of Mesoamerican origin) [12]. In the face of the intensification of adverse effects of climate change, such as the increase in the occurrence of biotic and abiotic stresses, there is a growing demand for actions aimed at mitigating the loss of genetic variability of agricultural crops important for food security. Studies related to plant genetic resources, such as studies involving the collection, characterization, and maintenance of germplasm banks, emerge as a promising strategy for the conservation of important species, such as the common bean (*P. vulgaris*). The creation and maintenance of germplasm banks are strategies that allow for the risks of genetic erosion to be reduced as well as for traits of resistance and/or tolerance to stresses that have been lost with the exploitation of these genetic resources to be rescued. In this sense, acting through genetic and plant breeding to develop more productive and resilient varieties in scenarios of environmental stresses is only possible when there is prior knowledge of the existing variability in each region.

Moreover, according to data from the National Supply Company (CONAB), in the 2022/2023 harvest period, Espírito Santo ranked 12th in productivity among the states producing the common bean, with a planted area of 8.9 thousand hectares, ranking 20th in national production. In 2005, the state ranked 13th in production [13]. Currently, the Southeast region (Minas Gerais, São Paulo, Rio de Janeiro, and Espírito Santo) accounts for almost 25% of national production [9]. Therefore, the reduction in production in the state could jeopardize national common bean production. The decrease recorded in production in the past 20 years may be related to several factors, among which the limited access of producers (mostly characterized by family farming) to cultivars more adapted to the climate of the cultivation regions stands out. To the best of our knowledge, only one study aimed at characterizing the germplasm of Espírito Santo, which was conducted by Fonseca et al. [13]. Therefore, there is an urgent need for the collection and characterization of common bean germplasm for the preservation of the species’ genetic resources, ensuring the availability of varieties adapted to family farming in Espírito Santo.

Most of the bean agrobiodiversity is conserved on farms, where farmers themselves conserve, select, and multiply their seeds year after year, ensuring their autonomy and income generation [5,10]. Therefore, the characterization of this germplasm, in which many genotypes are being lost, is of great importance. Morphological characterization is considered the most accessible way to characterize a genotype as it is important for understanding its potential and proper use [14]. ISSR molecular markers are efficient for studies of genetic variability because they are highly polymorphic, have good reproducibility, and require small amounts of DNA [15,16,17].

Considering the socio-economic importance of the common bean, it is essential to characterize the accessions to distinguish the genetic dissimilarity among them and identify parents for breeding programs. The characterization and preservation of germplasm collections contribute to the promotion of food security in the country, as advocated by the 2030 Agenda, to which Brazil is a signatory. Therefore, the objective of this work was to evaluate the genetic dissimilarity among traditional accessions of the common bean using morphological descriptors and molecular markers.

## 2. Results and Discussion

### 2.1. The Phenotypic Diversity among 67 Common Bean Accessions

**Figure 1:**
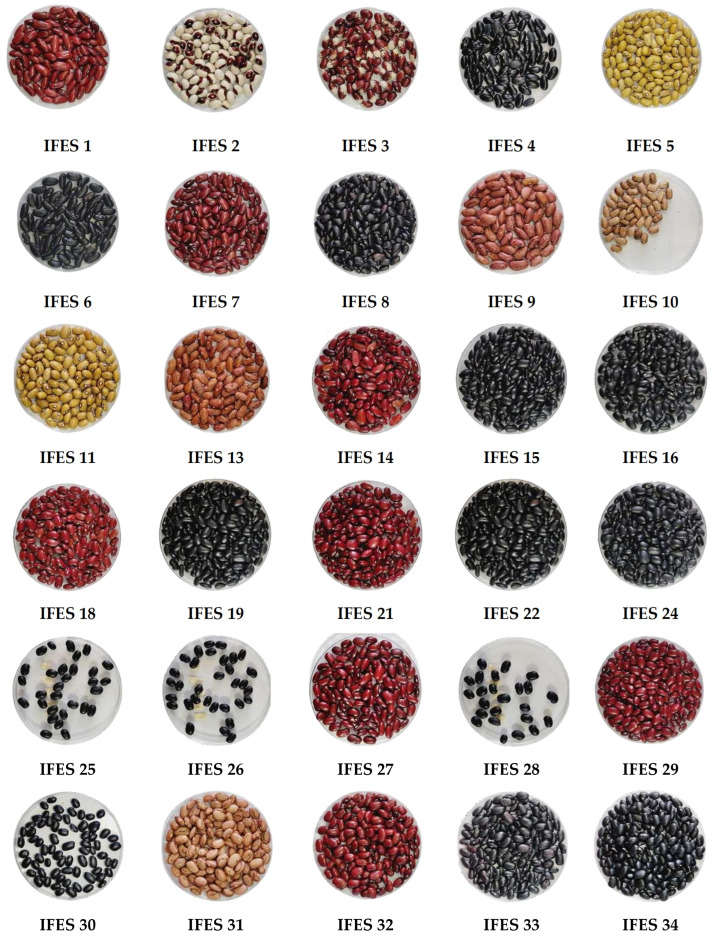

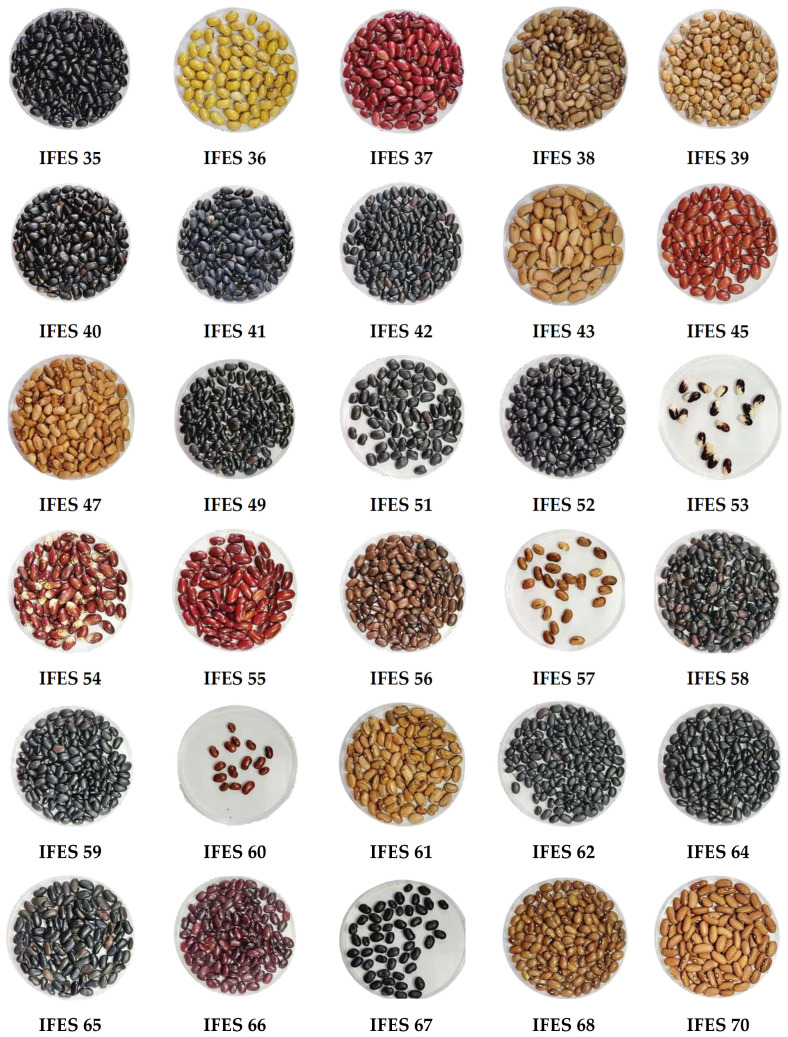

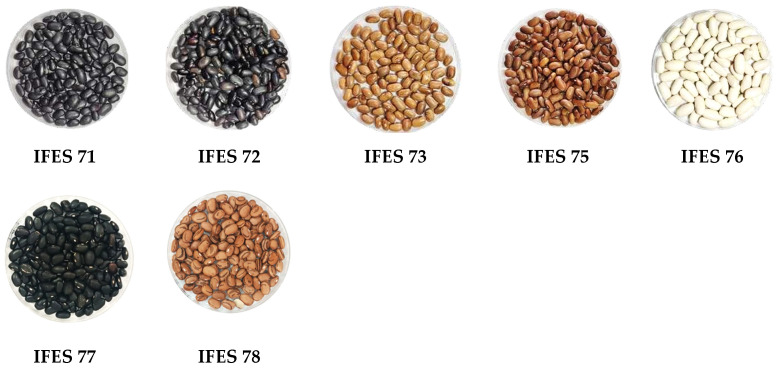
Phenotypic diversity of common bean (*P. vulgaris* L.) accessions belonging to Ifes—Campus de Alegre germplasm bank.

Based on the above considerations, the development of this study was deemed necessary. We describe the collection and characterization of 67 common bean accesses collected from various locations in the state of Espírito Santo. Based on qualitative and quantitative morphological descriptors and molecular characterization with 23 ISSR primers, we estimated the diversity of the collected accessions for incorporation into the bean germplasm bank, located at the Federal Institute of Education of Espírito Santo—Campus de Alegre, Alegre, ES. Based on morpho-agronomic characterization, we found a great phenotypic variability among the common bean accessions, with different shapes, sizes, weights, and colors for the grain (Figure 1), pod, flowers, and leaves. Plant breeders aiming to develop superior cultivars of a given species always rely on the existence and amount of genetic diversity accessible in the germplasm collection to be studied [18]. Nasar et al. [19] emphasized that local accessions exhibit considerable morphological diversity, reflecting the rich germplasm diversity due to geographical characteristics and microclimatic variations in bean-producing areas. In studies using morphological characterization, the largest possible number of descriptors should be used to obtain a more effective and complete analysis of dissimilarity [14]. In this study, apart from molecular characterization, 25 morphological descriptors were used to assess the genetic diversity among the studied genotypes, which allowed for the accessed characteristics to be grouped into several distinct classes.

For the quantitative characters, a significant difference was found between the accessions for all evaluated descriptors (Table 1), indicating variability among the tested genotypes. The coefficient of variation ranged from 3.69 to 17.48%, demonstrating good consistency in the experimental data. It is possible to infer that the present study had high experimental precision, as out of the 12 quantitative descriptors, 10 had low coefficients of variation (CVs) (less than 10%), and only two descriptors had medium CVs (10 to 20%) regarding the number of pods (NPP) and number of seeds per pod (NSP). It is possible that the fact that the experiment was conducted in a greenhouse contributed to the low CVs. Silveira et al. [20], when studying the genetic diversity of 100 bean landraces in Rio Grande do Sul, found CV values ranging from 17% to 22% for several descriptors common to this study.

In a study conducted by Rana et al. [21], which aimed to characterize more than 4000 accessions in India, the average weight of 100 seeds observed was 27.5 g, while the width and length of the seeds were 6.5 mm and 12 mm, respectively. The number of seeds per pod observed by the authors was 5.1, while the average leaflet length was 11.5 cm. The values observed by Rana et al. [21] are very close to those obtained in our study. In both studies, the evaluated germplasm is mainly composed of local varieties (originating from family farming) with few accessions of commercial origin. It is also noteworthy that most of the accessions studied by Rana et al. [21] are of Andean origin, which have larger and heavier seeds, unlike our work in which most of the accessions are of Mesoamerican origin, justifying smaller seed sizes and weights.

After the analysis of variance, the means of the morphological data were subjected to Scott-Knott mean clustering (*p* < 0.01, Table 2). The descriptor weight of 100 seeds presented the largest number of classes—ten in total—with values ranging from 10.04 g (IFES 72) to 37.30 g (IFES 55). In Brazil, there is a preference for smaller beans, which explains the lower weights of 100 seeds typically found for Mesoamerican beans. Nasar et al. [19] highlighted that the apparent preference for small-seeded accessions can be attributed to characteristics such as flavor, seed color, and cooking time. These preferences were also observed in various other parts of the world, including China [22] (Lei et al., 2020), India [23], Pakistan [19], and Italy [24]. In a study conducted by Santos et al. [25] aimed at evaluating the genetic variability of 49 common bean accessions of Andean origin (including traditional and improved varieties) belonging to the germplasm bank of the Federal University of Santa Maria (UFSM), higher values were observed for the weight of 100 seeds (22.32 g to 45.67 g).

The seed height was the second variable that encompassed the largest number of groups, with a total of nine classes, ranging from 3.53 mm (IFES 41) to 6.62 mm (IFES 16). The seed length and apex length characteristics each comprised eight groups. For the first characteristic, the values varied between 8.98 mm (IFES 41) and 17.53 mm (IFES 43), and for the apex length, they ranged from 6.17 mm (IFES 30) to 26.41 mm (IFES 01). The plant height variable is presented in seven classes, with values ranging from 34.38 cm (IFES 45) to 75.21 cm (IFES 18). In a study on the analysis of the genetic divergence of 22 common bean cultivars, focusing on characteristics related to plant architecture, plant height values close to those of the present work were obtained with an overall average of 57.06 cm [26]. This is an important descriptor for plant breeding that is related to plant stature and directly linked to harvestability. In this sense, most accessions in the present research have sizes similar to those of commercial cultivars.

The morphological descriptors of seed width and number of pods each comprised six classes. The seed width varied from 5.10 mm (IFES 41) to 8.13 mm (IFES 01), and the number of pods ranged from 2.96 (IFES 01) to 23.54 (IFES 26). The average number of pods per plant in the present study was 9.17. Cabral et al. [27] (2011a), who estimated the genetic variability of 57 traditional bean accessions, found an average of 8.16 pods per plant. In contrast, Kläsener et al. [26] (2022), who worked exclusively with commercial cultivars, reported an average of 15.58 pods per plant. This indicates that the genotypes analyzed in their research have undergone an extensive selection process for this characteristic.

The descriptors including the number of seeds per pod, pod length, pod width, leaf length, and leaf width had four classes each. The number of seeds per pod ranged from 3.21 (IFES 21) to 7.38 (IFES 65); pod length ranged from 81.86 mm (IFES 58) to 117.32 mm (IFES 06); pod width ranged from 9.01 mm (IFES 65) to 13.59 mm (IFES 45); leaf length ranged from 8.79 cm (IFES 40) to 15.71 cm (IFES 70); and leaf width ranged from 5.90 cm (IFES 29) to 11.21 cm (IFES 53). For the qualitative traits, significant morphological diversity was observed among the 67 common bean accessions with many phenotypic classes. For the flower standard color descriptor, white standard predominated (48%), followed by lilac (42%), white with lilac margins (7%), and purple (3%). In a study aiming to carry out the morpho-agronomic characterization of common bean accessions, India Jan et al. [28] (2021) described the predominance of white standards (40%), followed by lilac (33%), pink (17%), purple (6%), yellow (3%), and red (1%) standards. In a study conducted by Stoilova et al. [29] in which 30 common bean accessions from Bulgaria and Portugal were characterized, 70% of the genotypes presented lilac standards, and 30% were white.

For the seed brightness descriptor, 48% of the seeds were classified as dull, 34% had medium brightness, and 18% were shiny. Regarding seed brightness, Meza et al. [30], in their work on morphological characterization, described 27% of seeds as dull, 3.6% as medium, and 69% as shiny. Dull seed brightness, also known as opaque brightness, may be associated with poor genotype quality regarding cooking. This occurs due to the influence of the Asp gene, which alters the seed coat structure and hinders water absorption [31].

Regarding the seed shape, 38.6% were truncated and elongated, 28.4% cuboid, 15% kidney-shaped, 12% oval, and 6% round. Oval-shaped grains are the most accepted among Brazilian consumers; in most cases, grains with a flat kidney shape are more likely to be rejected [31]. Jan et al. [28], when characterizing 109 *Phaseolus vulgaris* accessions, mainly from the Jammu and Kashmir regions (India), reported that 28% were cuboid, 28% were rounded and oval, 27% were kidney-shaped, 16% were round, and 1% were oval. As for the seed coat pattern descriptor, it was absent in 79.1% of the grains, striped in 14.9%, marginal in 1.49%, and bicolor in 4.49%.

To better understand the genetic distance between accessions, the dissimilarity matrix was used through the Gower algorithm, which showed that, based on the morpho-agronomic descriptors used, the evaluated genotypes have an average distance of 0.57 (±0.07). The Gower dissimilarity matrix showed that accessions IFES 18 and IFES 45 are the most distant with a distance of 1.10. On the other hand, IFES 25 and IFES 67 were considered the closest accessions with a distance of 0.09. The high average distance between the accessions studied in this work indicates the high level of genetic diversity of the genotypes obtained. A greater dissimilarity is important in works where crossing is carried out with the aim of recombining parental characteristics as well as promoting greater segregation in a breeding program, increasing the possibility of selecting superior individuals in segregating generations [32,33].

**Figure 2:**
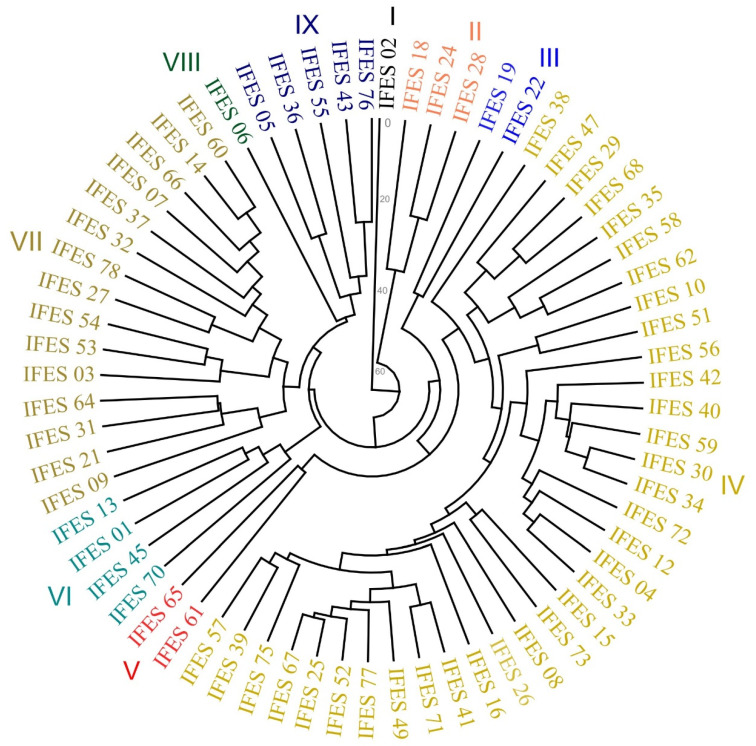
A dendrogram of genetic dissimilarity created using the Gower distance, based on quantitative and qualitative descriptors, for the 67 common bean accessions (cophenetic correlation = 0.86). The numbers I, II, III, IV, V, VI, VII, VIII, and IX refer to groups that include the genetically closest accessions.

The genetic distances between accessions were used to construct a dendrogram, grouped by the UPGMA method, which is presented in Figure 2. The UPGMA technique was efficient in fitting the cophenetic matrix to the dissimilarity matrix based on the generalized Mahalanobis distance with a cophenetic correlation of 0.86, demonstrating the high reliability of the grouping. It is estimated that there was a good fit between the distances, as according to Sokal and Rohlfe [34], an appropriate fit is evaluated by cophenetic correlation values greater than 0.80. Cabral et al. [26] reported the high precision of UPGMA clustering by the dissimilarity obtained by the generalized Mahalanobis distance in relation to other measures of dissimilarity.

Group I was composed of only accession IFES 2, which stood out for having white grains with purple spots, wider pods, and a shorter pod length. Group II gathered three accessions (IFES 18, IFES 24, and IFES 28) characterized by tall plant heights, a small grain size, and a low number of seeds per pod. Group III consisted of two accessions (IFES 19 and IFES 22) that have an intensely shiny black grain color, a cuboid shape, an indeterminate growth habit, and a lower pod apex length. This characteristic tends to be beneficial in many regions of Brazil because beans are often grown in association with maize, and thus, the bean plant can lean on the maize plant as it grows. Kläsener et al. [26] emphasized that an indeterminate growth habit makes harvesting and cultural practices difficult; however, it contributes to maintaining the quality of the grains produced on a large scale as the pods do not touch the soil. Nasar et al. [19] described an indeterminate habit as an ecophysiological adaptation to achieve maximum light exposure and increase its photosynthetic efficiency.

Group IV was the largest group, consisting of 34 accessions, formed mostly by beans with a black and beige grain color (‘carioca’ type; 89%); in other words a large part of the beans in this group were of Mesoamerican origin. The commercial variety, IFES 77 (black bean), was allocated in this group. Additionally, this group stood out for presenting a tegument pattern that varied only between a striped (common in beige-colored varieties) and absent pattern (common in black-colored varieties). Group V was formed by accessions IFES 61 and IFES 65, which stood out for having a high number of seeds per pod. IFES 61 (carioca bean) and IFES 65 (black bean) are from the same genetic pool, Mesoamerican, and presented a purple standard color. These accessions have the potential to be used in breeding programs since a higher number of seeds produced in the pods was observed. The characteristic number of seeds per pod is of great importance in bean productivity; in general, genotypes with high values for this characteristic are selected for breeding programs focusing on increasing crop production [29]. Yield is the main characteristic focused on in breeding programs; however, this characteristic is very complex as it involves a significant number of genes, which act directly or indirectly on the grain yield [35].

Group VI was constituted by four accessions (IFES 1, IFES 13, IFES 45, and IFES 70) that presented a large grain size, characteristic of the Andean gene pool, a reniform shape, and a low number of pods per plant. Group VII was formed by seven accessions that presented a dorsal apex orientation of the pod and seeds with variations in red color. It is noteworthy that in this group, beans from the Andean pool were also concentrated with a red or reddish-brown phenotype, including the commercial variety IFES 21 (red bean).

Group VIII allocated only accession IFES 6, characterized by black grains, long lengths of the leaflet and pod, and an average weight of 25.6 g for 100 seeds. The seed width variable was relatively low, and this, coupled with the fact that the seed length variable presented a relatively high average, allows us to infer that the seeds of accession IFES 6 have a more elongated phenotype and are not very thick. This phenotype, according to Rana et al. [21], may be associated with a characteristic of great importance for the consumer market, which is the short cooking time of the beans. In several states of Brazil, consumers prefer bean varieties with a black tegument color and with an average seed weight ranging from 25 to 40 g per 100 seeds, which are characteristics present in accession IFES 6; thus, this genotype has great potential for use in the market [36].

Group IX gathered five accessions (IFES 5, IFES 36, IFES 43, IFES 55, and IFES 76), which are those that have the most discrepant grain color phenotypes, being yellow and white, classified as being of Andean origin. In addition, they have longer leaflet lengths and a heavier 100-seed weight, a low number of seeds per pod, a white standard color with lilac margins, an ovate bracteole shape, and no tegument pattern. Nasar et al. [19] described that Andean varieties exhibit larger leaves, which is attributed to a selection pressure caused by colder temperatures, which inversely affect the leaf size.

In both quantitative and qualitative characterization, no duplicates were found in the material under study, which suggests the importance of keeping all accessions in the germplasm bank since they represent a valuable source of genetic diversity that may be useful for plant breeding in the future [32].

### 2.2. ISSR Characterization and Molecular Diversity

Molecular characterization involved screening with 48 ISSR primers, of which 23 were selected for producing greater polymorphism and band sharpness. A total of 146 (92.4%) polymorphic bands were obtained with the 23 selected primers, as shown in Table 3. The average number of polymorphic fragments produced per primer was 6.35. The primer that generated the highest number of polymorphic bands was A28 with 11 bands, and the primers P19 and P21 generated 3 bands each, being the smallest polymorphisms.

A high level of polymorphism was found using ISSR primers, proving that it is an efficient technique to access the genetic variability of common bean genotypes, as described in other studies with the same crop [14,37,38]. Hamouda et al. [14] highlighted that the high level of variability found in common bean accessions may be associated with the different origins of the beans (Andean or Mesoamerican) as well as selection and geographical and environmental factors, making the species an important genetic resource to be conserved and characterized in different parts of the world.

Sakhravi et al. [38], working with 41 bean accessions from Iran, obtained 172 polymorphic bands using 20 ISSR primers with an average of 6.88 polymorphic bands per primer. Hamouda et al. [14], studying 12 common bean accessions from Egypt, found 69.1% polymorphism, and Dagnew et al. [37], working with 12 common bean accessions from Ethiopia, obtained a total of 69 polymorphic bands with an average of 9.85 polymorphic bands per primer. All the above results are in line with the data obtained in the present study in which 67 bean accessions were characterized with 23 ISSR primers, resulting in 146 polymorphic bands or 6.35 polymorphic bands per primer.

**Figure 3:**
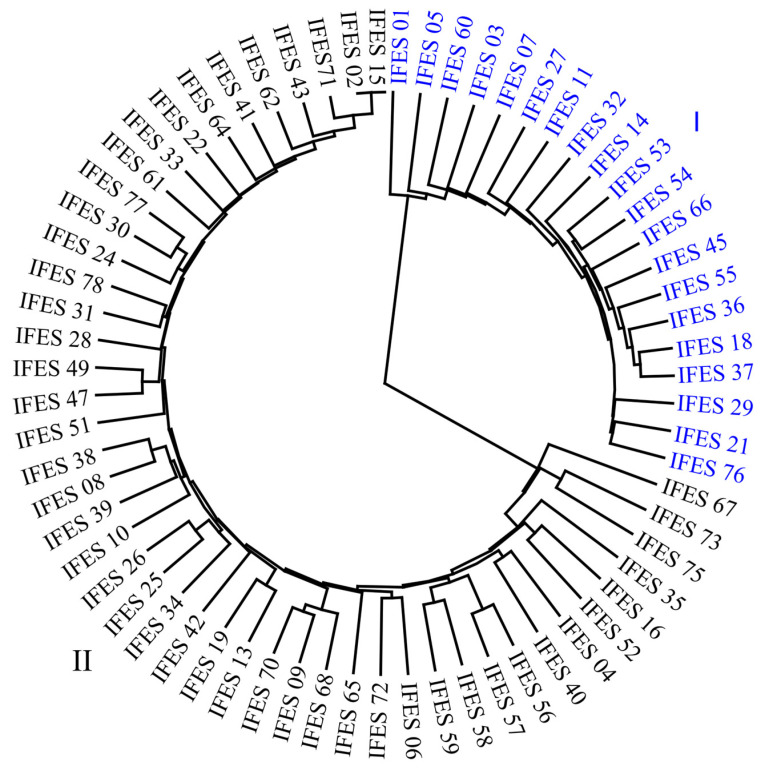
The dendrogram obtained with UPGMA from the Jaccard dissimilarity matrix of 67 accessions of the common bean based on 146 polymorphic ISSR markers (cophenetic correlation = 0.99). Numbers I and II refer to the groups that include the genetically closest accessions, separated according to gene pools: I—accessions of Andean origin; II—accessions of Mesoamerican origin.

The average distance observed between genotypes was 0.47 (±0.07). Using the Jaccard Index, it was possible to determine that the most distant genotypes were IFES 14 and IFES 51 with a distance of 0.71, while accessions IFES 02 and IFES 15 were considered the most similar with a distance of 0.01. The genetic distances between accessions were used to construct a dendrogram, grouped by the UPGMA method, which is presented in Figure 3. The UPGMA technique was efficient in fitting the distances, with a cophenetic correlation of 0.99 for the associations between the distance matrix and the dendrogram of molecular variables, which is considered very satisfactory according to Sokal and Rohlfe [34].

The dendrogram allowed for the genetic distinction of the individuals, resulting in separation into two main groups: group I, consisting of accessions of Andean origin, and group II, consisting of accessions of Mesoamerican origin. Thus, there is a high accuracy in establishing groups according to the genetic pools of the common bean. Group I contains 20 accessions and includes beans with larger sizes and red and cranberry coloration (beige with spots and other variations). Group II encompasses the remaining 47 genotypes, and within this group are smaller beans, popularly known as black, carioca, and brown beans. It is worth noting that the control IFES 21 (red bean), belonging to the Andean genetic pool, was allocated in group I, and controls IFES 77 (black bean) and IFES 78 (carioca bean), belonging to the Mesoamerican genetic pool, were allocated in group II, as expected. Nasar et al. [19], Nogueira et al. [33], and Cabral et al. [39], evaluating the diversity of common bean genotypes through molecular markers, also observed grouping according to the centers of origin.

Common bean breeding programs in Brazil mainly use Mesoamerican germplasm, highlighting the need to emphasize the introgression of Andean germplasm in germplasm banks and commercial cultivars of this species in the country, following the example of other tropical countries [11]. The dissimilarity observed between the accessions of the germplasm collection and the commercial cultivars studied reinforces the need for the introgression of this germplasm in common bean breeding programs.

The combined use of morphological data and ISSR molecular markers allowed for a detailed description of the variability present in our common bean collection and excluded the existence of duplicates in the collection. The data enable a choice regarding the potential of different accessions, which is important information for breeding programs and farmers as it allows for the selection of genotypes for crosses and meets different market niches, respectively. The importance of these markers used in the research is well documented in various studies [14,19,37,38,40].

## 3. Materials and Methods

### 3.1. Plant Material, Environmental Conditions, and Experimental Design

The experiment was conducted in a greenhouse in the Agroecology Sector of Instituto Federal do Espírito Santo (IFES)—Campus de Alegre, Alegre, Espírito Santo, Brazil (41°25′50″ S to 41°29′44″ W, 277 m above sea level). According to the Köppen classification, the study environment is classified as a humid subtropical climate with an average temperature of 23.1 °C and an annual precipitation of approximately 1341 mm [41]. Sixty-seven accessions of common bean were characterized, comprising the Active Germplasm Bank of Ifes, including sixty-four traditional accessions from family farming and three commercial ones used as controls, namely IFES 21, IFES 77, and IFES 78 (Table 4).

The experiment was arranged in a Randomized Complete Block Design (RCBD) with three replications. Each experimental unit consisted of a single row with five plants, totaling 1005 plants, with a spacing of 0.05 m between rows and 0.50 m between plants. Cultural practices and pest and disease control were performed following the recommendations for bean cultivation [42].

Before planting, two soil analyses were conducted, one physical and one chemical, to understand the nutritional status and fertility level of the area, allowing for the rational use of fertilizers. The analyses were carried out at the Soil Chemical Analysis Laboratory of the Federal University of Espírito Santo (UFES) in Alegre, ES, Brazil. Considering the chemical soil analysis and the recommendations of Prezotti [43] for bean cultivation, it was inferred that the area did not require the application of lime. Two fertilizations were applied in the area: the planting fertilization, which involved incorporating 50 kg ha^−1^ of NPK 20-05-20, and the topdressing, performed 30 days after planting by applying 200 kg ha^−1^ of NPK 20-05-20 fertilizer. The topdressing was split into two applications of 100 kg ha^−1^ of fertilizer with a 15-day interval between each topdressing.

After 90 days of planting, the common bean accessions were characterized based on specific morphological descriptors for *Phaseolus vulgaris* L. developed by the International Board for Plant Genetic Resources [44]. A total of 25 morphological traits were evaluated, of which 12 were quantitative traits (Table 5) and 13 were qualitative traits (Table 6).

### 3.2. Statistical Analysis

The quantitative data were submitted to an analysis of variance (ANOVA) using the F test (*p* < 0.01). After identifying significant effects for the source of variation genotypes, the Scott-Knott clustering test was used at 1% significance level grouping at the univariate level. The genotype mean of each trait was estimated using the following equation:

$$µ=\sum_{i=1}^{n}x_{i}/n$$

where µ is the mean; *x_i_* is the value observed in the *i*-th plot; and *n* is the number of observations (replications). The total variance (*σ*^2^) was estimated using the following equation:

$$\sigma^{2}=\sum_{i=1}^{n}{\left(x_{i}-µ\right)}^{2}/n\;$$

where *x_i_* is the mean of the *i*-th genotype; µ is the general mean; and n is the total number of genotypes. The coefficient of variation (CV) was estimated using the following equation:

$$CV\left(\%\right)=\sqrt{\sigma^{2}}*100/µ$$

where µ is the general mean of the experiment. The qualitative descriptors were submitted to a descriptive statistical analysis. For the elaboration of the dendrogram of quantitative and qualitative data, Pearson’s correlation and the Mahalanobis Generalized Distance were employed. For the dendrogram, the UPGMA method was used to group the accessions. The statistical analyses were performed using the RStudio software version 2024.04.2+764 [45], and the results were validated by calculating the cophenetic correlation coefficient (CCC).

### 3.3. Molecular Characterization

For DNA extraction, young leaves of each accession were wrapped in aluminum foil, labeled, and immediately immersed in liquid nitrogen to avoid DNA degradation. In the lab, this material was macerated in liquid nitrogen to the consistency of a fine powder. Approximately 100 mg of macerated tissue was transferred to 2.0 mL tubes and immersed in liquid N2 for DNA extraction using the protocol of Doyle and Doyle [46] with modifications. DNA quantification was performed in 1.0% agarose gels; the concentrations of the markers were measured with the Image program using a 250 bp marker as a standard for comparison. The DNA was diluted to a final concentration of 5 ng µL^−1^.

PCR amplification reactions were performed according to the protocol used by Williams et al. [47] with some modifications in an Applied Biosystems thermocycler. The final reaction volume was 19 µL and contained the following reagents: 10 mmol L-1 of Tris-HCl, pH of 8.3, 50 mmol L^−1^ of KCl, 2.4 mmol L^−1^ of MgCl_2_, 100 μmol L^−1^ of each dNTP, 0.4 μmol L^−1^ of oligonucleotide primers, 5 ng of genomic DNA, and 0.75 units of Taq DNA polymerase. A 2 μL DNA aliquot was placed in each tube, and the above ingredients were added.

The PCR reactions (GeneAmp PCR System 9700 Thermal cycler, Applied Biosystems, Waltham, MA, USA) were run as follows: 3 min. at 94 °C for the initial denaturation, followed by 40 cycles consisting of 94 °C for 1 min., 45 °C or 49 °C for 1 min. (depending on the primer), and 72 °C for 3 min., and a final extension step of 72 °C for 7 min. The amplified fragments were separated in 1.5% agarose gel, stained with gel red, and visualized under a UV light (photodocumented with Minibis Pro, Bio-imaging System). The amplification conditions were optimized for each primer to determine the best temperature for amplification. We used 23 ISSR primers (UBC primers, Vancouver, BC, Canada) (Table 7).

A statistical analysis of the data was performed by considering a binary matrix that was constructed using the value of ‘1’ to indicate a present band and ‘0’ to indicate an absent band. The monomorphic bands were eliminated. The binary data were submitted to analysis using the Genes program [48], and a dendrogram was constructed using the R software version 2024.04.2+764 [45]. Clustering was performed using the Unweighted Paired Group Method using Arithmetic averages (UPGMA) based on the Jaccard Index, and the results were validated by the cophenetic correlation coefficient (CCC).

## 4. Conclusions

The morphological and molecular analyses were efficient in assessing the genetic variability among the common bean accessions, revealing a high variability. This suggests that the morpho-agronomic descriptors were able to detect the diversity present among the accessions. The diversity found is important information for breeding programs and highlights the importance of conserving this genetic resource. Additionally, no genetic duplicates were found among the accessions in the collection.

The molecular analysis allowed for a more precise grouping of the accessions using the UPGMA method, grouping them according to the gene pools: Andean and Mesoamerican. The results of this study will help fill the gap in the knowledge about the variability in *P. vulgaris* in the state of Espírito Santo and will allow the establishment of a germplasm collection that will serve as a basis for the improvement of the species in the different regions where the accessions were obtained. Ultimately, we hope these results will contribute to promoting the use of these resources and ensuring food security in line with the objectives of the UN’s 2030 Agenda.

## Figures and Tables

**Table 1:** Analysis of variance of 12 quantitative morphological descriptors evaluated in 67 common bean accessions belonging to Active Germplasm Bank of Ifes—Campus de Alegre, ES, Brazil.

Traits	Mean Squares			Mean	CV%
	Genotype	Block	Error		
PH	180.42 **	18.17	12.597	50.27	7.06
LL	6.05 **	1.04	1.0382	12.19	8.36
LW	3.66 **	0.02	0.3786	8.57	7.18
PW	2.86 **	0.21	0.28848	11.09	4.84
PL	186.59 **	55.31	27.066	98.30	5.29
AL	46.92 **	3.10	0.85	10.57	8.72
NPP	42.38 **	0.94	2.567	9.17	17.48
SL	9.03 **	1.55	0.1825	11.57	3.69
SW	1.50 **	0.04	0.08163	6.48	4.41
SH	1.35 **	0.05	0.03812	4.98	3.92
NSP	2.07 **	0.71	0.38812	4.98	12.52
W100S	89.05 **	2.73	1.671	21.43	6.03

PH—plant height (cm); LL—leaflet length (cm); LW—leaflet width (cm); PW—pod width (mm); PL—pod length (mm); AL—apex length (mm); NPP—number of pods per plant (un); SL—seed length (mm); SW—seed width (mm); SH—seed height (mm); NSP—number of seeds per pod (un); W100S—weight of 100 seeds (g). ** denote significance at the 5% level according to the F test.

**Table 2:** Grouping of means using Scott-Knott test * (*p* < 0.01) for 12 morphological quantitative traits evaluated in 67 common bean accessions belonging to Ifes—Campus de Alegre, ES, Brazil.

Accession	PH	LL	LW	PW	PL	AL	NPP	SL	SW	SH	NSP	W100S
IFES 1	12.75 b	9.81 b	51.25 e	91.82 c	10.93 c	26.41 a	2.96 f	16.15 b	6.21 b	8.13 a	5.17 b	29.56 c
IFES 2	11.71 c	7.10 d	47.96 e	74.25 e	13.00 a	10.50 f	8.67 d	8.99 h	5.65 c	7.35 b	4.04 d	19.60 f
IFES 3	12.73 b	8.27 c	43.67 f	99.66 b	11.66 b	18.40 b	7.83 d	13.93 c	5.87 c	7.08 c	5.58 b	30.57 c
IFES 4	10.54 d	8.44 c	58.96 c	98.21 c	10.72 c	9.79 f	5.92 e	11.21 e	5.95 b	6.54 d	6.83 a	17.74 g
IFES 5	14.50 a	11.15 a	41.63 f	99.68 b	10.40 d	12.24 e	7.33 d	10.41 f	5.55 d	7.11 c	4.25 d	24.47 e
IFES 6	14.42 a	9.19 b	43.96 f	117.32 a	12.10 b	19.98 b	5.21 e	12.08 e	4.93 e	5.60 f	5.96 b	25.60 d
IFES 7	14.08 a	7.40 d	46.38 e	101.10 b	10.24 d	8.26 g	12.38 c	11.13 f	4.69 f	6.16 e	4.42 c	23.99 e
IFES 8	10.44 d	6.65 d	51.88 e	97.00 c	12.11 b	9.33 f	16.07 b	11.65 e	5.03 e	6.55 d	6.38 a	20.15 f
IFES 9	12.79 b	8.02 c	58.96 c	107.53 a	12.75 a	19.12 b	3.75 f	13.74 c	5.09 e	7.40 b	3.38 d	25.11 d
IFES 10	13.58 b	9.21 b	45.54 f	105.32 b	10.87 c	8.05 g	4.50 e	10.70 f	4.43 g	6.24 e	4.42 c	17.44 g
IFES 12	13.27 b	8.90 b	57.50 c	96.15 c	12.33 b	9.10 f	9.75 c	11.29 e	4.18 g	5.65 f	5.42 b	19.43 f
IFES 13	12.75 b	8.38 c	47.21 e	96.68 c	11.98 b	14.58 d	3.96 f	14.16 c	5.50 d	6.86 c	4.17 d	27.53 d
IFES 14	12.54 b	8.48 c	51.38 e	99.50 b	10.73 c	8.55 g	10.42 c	12.56 d	5.35 d	6.92 c	5.42 b	24.40 e
IFES 15	10.56 d	8.02 c	37.50 g	93.99 c	10.54 c	13.75 d	10.32 c	9.88 g	4.04 h	5.37 f	5.54 b	13.96 i
IFES 16	11.94 c	8.13 c	48.00 e	93.45 c	10.90 c	9.17 f	7.17 d	16.09 b	6.62 a	8.10 a	6.00 b	20.23 f
IFES 18	11.04 c	8.04 c	75.21 a	117.15 a	11.41 c	10.57 f	8.17 d	10.68 f	4.34 g	6.20 e	6.04 b	19.55 f
IFES 19	12.38 b	7.04 d	50.42 e	112.40 a	11.70 b	8.66 g	9.38 c	11.08 f	5.11 e	6.49 d	5.21 b	20.05 f
IFES 21	12.17 c	7.96 c	55.82 d	103.83 b	12.86 a	10.63 f	11.08 c	10.72 f	5.54 d	6.79 c	3.21 d	24.02 e
IFES 22	14.63 a	10.44 a	52.08 e	95.75 c	10.60 c	8.88 g	12.21 c	10.12 g	3.94 h	5.53 f	5.25 b	18.14 g
IFES 24	11.00 c	7.79 c	70.21 a	104.81 b	13.43 a	7.75 g	8.46 d	10.57 f	4.16 g	5.76 f	6.04 b	16.01 h
IFES 25	12.88 b	8.96 b	48.33 e	99.64 b	11.29 c	9.38 f	9.50 c	10.89 f	4.25 g	5.91 e	4.96 c	18.38 g
IFES 26	10.56 d	8.46 c	48.75 e	94.04 c	10.93 c	9.00 f	23.54 a	10.78 f	4.26 g	6.34 e	4.83 c	17.72 g
IFES 27	13.50 b	8.00 c	42.46 f	105.65 b	10.56 c	8.13 g	10.19 c	11.63 e	5.03 e	6.58 d	4.96 c	22.98 e
IFES 28	11.13 c	9.71 b	67.29 b	105.77 b	10.63 c	7.60 g	6.92 d	13.26 c	5.84 c	5.74 f	3.67 d	16.78 h
IFES 29	10.54 d	5.90 d	43.63 f	91.61 c	11.64 b	7.56 g	5.63 e	11.24 e	4.81 f	6.60 d	5.04 b	20.65 f
IFES 30	12.60 b	7.98 c	53.96 d	93.04 c	9.80 d	6.17 h	8.54 d	9.80 g	4.39 g	6.23 e	4.88 c	18.01 g
IFES 31	11.21 c	8.33 c	56.92 d	112.93 a	12.23 b	9.45 f	12.21 c	11.86 e	4.89 e	7.03 c	5.17 b	21.29 f
IFES 32	12.35 b	8.79 c	63.96 b	99.56 b	10.92 c	9.23 f	10.63 c	11.58 e	4.84 f	6.71 d	4.83 c	23.07 e
IFES 35	11.83 c	8.13 c	43.96 f	84.71 d	9.49 d	13.10 e	7.42 d	11.96 e	5.36 d	7.13 c	5.54 b	21.28 f
IFES 36	13.54 b	9.27 b	44.04 f	100.95 b	11.24 c	16.56 c	6.21 d	12.85 d	6.25 b	7.62 b	4.67 c	26.41 d
IFES 37	12.04 c	8.52 c	49.67 e	93.00 c	10.51 c	6.68 h	15.08 b	11.66 e	5.43 d	6.39 e	5.42 b	19.82 f
IFES 38	12.04 c	10.90 a	39.17 g	97.48 c	10.15 d	9.38 f	14.75 b	12.85 d	5.60 c	6.84 c	4.79 c	15.21 i
IFES 39	13.33 b	7.73 c	46.71 e	98.86 b	11.38 c	5.59 h	11.13 c	10.96 f	4.94 e	6.66 d	5.00 c	19.66 f
IFES 40	8.79 d	9.10 b	55.38 d	86.80 d	10.06 d	11.63 e	8.25 d	10.10 g	4.70 f	6.05 e	4.42 c	16.79 h
IFES 41	11.90 c	7.60 c	47.71 e	96.27 c	10.47 c	7.49 g	10.50 c	8.98 h	3.53 i	5.10 f	5.67 b	19.27 f
IFES 42	13.38 b	9.32 b	54.38 d	100.67 b	11.48 b	8.29 g	7.58 d	9.20 h	3.95 h	5.50 f	5.00 c	14.74 i
IFES 43	14.98 a	10.92 a	43.04 f	107.59 a	9.78 d	17.20 c	6.00 e	17.53 a	5.45 d	7.93 a	4.25 d	33.32 b
IFES 45	12.50 b	8.48 c	34.38 g	92.30 c	13.59 a	14.92 d	6.33 d	13.31 c	6.47 a	7.91 a	4.00 d	28.58 c
IFES 47	9.96 d	7.54 c	44.79 f	89.53 d	10.61 c	8.62 g	5.17 e	9.66 g	4.93 e	6.05 e	5.63 b	17.34 g
IFES 49	11.96 c	8.98 b	45.21 f	100.22 b	10.73 c	10.16 f	7.75 d	10.68 f	4.51 g	6.12 e	5.17 b	15.52 h
IFES 51	13.02 b	8.33 c	47.92 e	103.86 b	10.78 c	9.76 f	4.46 e	10.12 g	4.35 g	5.76 f	4.96 c	13.37 i
IFES 52	11.65 c	8.00 c	47.71 e	102.37 b	10.93 c	9.32 f	12.63 c	10.58 f	4.09 h	6.60 d	5.25 b	18.57 g
IFES 53	15.13 a	11.21 a	54.00 d	105.60 b	12.62 a	15.57 d	11.07 c	11.32 e	5.37 d	6.98 c	4.42 c	29.98 c
IFES 54	12.75 b	9.38 b	46.25 e	112.75 a	10.74 c	16.53 c	11.71 c	13.07 d	5.60 c	6.97 c	4.04 d	30.79 c
IFES 55	13.04 b	7.79 c	43.79 f	99.86 b	12.23 b	18.79 b	16.08 b	13.05 d	4.99 e	7.13 c	3.63 d	37.30 a
IFES 56	10.38 d	9.52 b	55.32 d	97.25 c	11.30 c	9.93 f	1.92 f	11.13 f	5.04 e	6.66 d	4.46 c	16.90 h
IFES 57	11.13 c	7.90 c	44.38 f	96.49 c	12.40 b	9.57 f	10.32 c	11.21 e	5.02 e	6.44 d	4.79 c	19.92 f
IFES 58	11.50 c	8.67 c	48.54 e	81.86 d	10.72 c	6.91 h	10.67 c	9.38 h	4.97 e	5.88 e	5.46 b	19.48 f
IFES 59	12.08 c	9.00 b	54.38 d	93.35 c	10.67 c	7.81 g	5.46 e	10.10 g	4.69 f	5.74 f	5.75 b	14.87 i
IFES 60	12.27 b	9.40 b	53.83 d	94.48 c	10.08 d	9.64 f	9.50 c	12.91 d	4.87 e	6.74 d	3.83 d	25.69 d
IFES 61	10.48 d	7.63 c	57.42 c	88.19 d	12.10 b	11.73 e	11.71 c	12.38 d	4.65 f	5.79 f	5.63 b	24.19 e
IFES 62	12.77 b	8.02 c	44.83 f	88.13 d	10.15 d	7.23 h	13.88 b	10.31 g	4.35 g	5.94 e	4.92 c	18.93 g
IFES 64	11.83 c	8.48 c	61.46 c	109.18 a	12.37 b	9.13 f	13.71 b	10.30 g	4.35 g	6.85 c	5.58 b	24.21 e
IFES 65	12.54 b	11.15 a	54.38 d	83.26 d	9.01 d	8.49 g	5.75 e	12.37 d	5.34 d	5.63 f	7.38 a	21.00 f
IFES 66	10.10 d	7.69 c	44.17 f	97.88 c	10.08 d	8.35 g	9.25 d	12.98 d	5.55 d	7.62 b	4.25 d	26.27 d
IFES 67	13.46 b	9.27 b	49.79 e	100.27 b	11.91 b	9.63 f	10.46 c	10.82 f	4.56 f	6.58 d	5.63 b	17.36 g
IFES 68	10.79 d	8.19 c	42.21 f	93.01 c	9.93 d	8.43 g	7.42 d	10.70 f	5.73 c	6.58 d	5.79 b	19.47 f
IFES 70	15.71 a	10.66 a	62.19 c	93.90 c	10.71 c	8.64 g	2.96 f	13.55 c	5.31 d	6.92 c	4.21 d	29.51 c
IFES 71	12.46 b	8.85 b	48.13 e	93.27 c	10.64 c	8.16 g	11.42 c	10.75 f	4.41 g	5.48 f	4.83 c	19.43 f
IFES 72	11.75 c	8.21 c	60.32 c	99.04 b	11.03 c	7.81 g	7.46 d	9.21 h	4.08 h	5.68 f	5.79 b	10.04 j
IFES 73	8.94 d	7.50 c	41.67 f	96.55 c	9.88 d	6.89 h	8.21 d	10.50 f	5.05 e	6.42 d	3.42 d	18.65 g
IFES 75	11.50 c	8.08 c	46.25 e	100.90 b	9.75 d	8.45 g	11.57 c	13.50 c	4.67 f	6.52 d	5.25 b	17.66 g
IFES 76	11.77 c	6.77 d	41.54 f	103.84 b	10.10 d	16.81 c	7.83 d	13.84 c	5.55 d	6.87 c	3.38 d	35.95 a
IFES 77	13.42 b	8.44 c	50.21 e	101.17 b	10.39 d	8.37 g	12.75 c	10.71 f	4.30 g	5.89 e	4.79 c	21.03 f
IFES 78	13.44 b	7.60 c	44.58 f	99.48 b	11.23 c	9.65 f	14.19 b	12.62 d	5.98 b	7.17 c	5.96 b	25.89 d

PH—plant height (cm); LL—leaflet length (cm); LW—leaflet width (cm); PW—pod width (mm); PL—pod length (mm); AL—apex length (mm); NPP—number of pods per plant (un); SL—seed length (mm); SW—seed width (mm); SH—seed height (mm); NSP—number of seeds per pod (un); W100S—weight of 100 seeds (g). * Means followed by the same letter in the column indicate groups of similar accessions.

**Table 3:** Descriptions of the number of bands and the polymorphism rates for 23 ISSR primers applied to 67 common bean accessions.

Primer	Number of Bands	Number of Polymorphic Bands	Polymorphism Rate
A1	9	8	88.8%
A3	6	6	100%
A4	7	6	85.7%
A7	7	7	100%
A11	8	8	100%
A12	12	12	100%
A17	5	5	100%
A18	8	7	87.5%
A21	9	9	100%
A23	7	6	85.7%
A28	12	11	91.7%
P12	6	6	100%
P13	6	5	83.3%
P14	6	6	100%
P15	6	5	83.3%
P16	6	6	100%
P17	6	5	83.3%
P18	4	4	100%
P19	5	3	60%
P20	5	5	100%
P21	4	3	75%
P22	5	4	80%
P23	7	7	100%
Total	158	146	92.4%

**Table 4:** Passport information (identification, common name, and origin) of the 67 common bean accessions belonging to the Active Germplasm Bank of Ifes, Campus de Alegre.

Accession	Common Name	Origin
IFES 01	Feijão Vermelho	Cachoeiro de Itapemirim, ES
IFES 02	Feijão Beijinho de Moça	Colatina, ES
IFES 03	Feijão Bolinha	Bom Jesus do Itabapoana, RJ
IFES 04	Feijão Preto	Alegre, ES
IFES 05	Feijão Amarelo	Alegre, ES
IFES 06	Feijão 60 dias	Alegre, ES
IFES 07	Feijão Vermelho	Alegre, ES
IFES 08	Feijão Preto	Cachoeiro de Itapemirim, ES
IFES 09	Feijão Amendoim	Cachoeiro de Itapemirim, ES
IFES 10	Feijão Carioca	Bom Jesus do Itabapoana, RJ
IFES 12	Feijão Carioquinha	Alegre, ES
IFES 13	Feijão Amendoim	Alegre, ES
IFES 14	Feijão Abundância	Alegre, ES
IFES 15	Feijão Preto	Alegre, ES
IFES 16	Feijão Painã	Muniz Freire, ES
IFES 18	Feijão Vermelho	Muniz Freire, ES
IFES 19	Feijão Vagem Branca	Muniz Freire, ES
IFES 21	Feijão Vermelho	Comercial
IFES 22	Feijão Preto	Alegre, ES
IFES 24	Feijão Serrano	Vargem Alta, ES
IFES 25	Feijão Vagem Riscada	Vargem Alta, ES
IFES 26	Feijão Vagem Branca	Vargem Alta, ES
IFES 27	Feijão Roxinho	Vargem Alta, ES
IFES 28	Feijão Vagem Preta	Vargem Alta, ES
IFES 29	Feijão Vermelho	Muniz Freire, ES
IFES 30	Feijão Fava Preta	São Francisco de Itabapoana, RJ
IFES 31	Feijão Carioquinha	Colatina, ES
IFES 32	Feijão Vermelho	Colatina, ES
IFES 33	Feijão Preto	Santa Maria de Jetibá, ES
IFES 34	Feijão Preto	Colatina, ES
IFES 35	Feijão Preto	Santa Maria de Jetibá, ES
IFES 36	Feijão Fradinho	Santa Maria de Jetibá, ES
IFES 37	Feijão Vermelho	Santa Teresa, ES
IFES 38	Feijão Mulatinho	Santa Maria de Jetibá, ES
IFES 39	Feijão Carioca Branco	Colatina, ES
IFES 40	Feijão Preto Comum	Porto Velho, RO
IFES 41	Feijão Preto	Aracruz, ES
IFES 42	Feijão Caetezinho	Aracruz, ES
IFES 43	Feijão Manteigão	Colatina, ES
IFES 45	Feijão Vermelho	Colatina, ES
IFES 47	Feijão Penquinha	Linhares, ES
IFES 49	Feijão Irapuru	Iúna, ES
IFES 51	Feijão Preto	Afonso Cláudio, ES
IFES 52	Feijão Vagem Branca	Iúna, ES
IFES 53	Feijão Palhacinho	Linhares, ES
IFES 54	Feijão Palhacinho	Domingos Martins
IFES 55	Feijão Manteigão Vermelho	Aracruz, ES
IFES 56	Feijão Nova Planta	Linhares, ES
IFES 57	Feijão Carioquinha	Alfredo Chaves, ES
IFES 58	Feijão Preto Comum	Colatina, ES
IFES 59	FeijãoIrapuru	Iúna, ES
IFES 60	Feijão Vermelho Escuro	Aracruz, ES
IFES 61	Feijão Carioquinha	Campos dos Goytacazes, RJ
IFES 62	Feijão Caetezinho	Colatina, ES
IFES 64	Feijão Painã	Iúna, ES
IFES 65	Feijão Preto	Alfredo Chaves, ES
IFES 66	Feijão Chamego	Afonso Cláudio, ES
IFES 67	Feijão Painã	Iúna, ES
IFES 68	Feijão Terrinha	Domingos Martins, ES
IFES 70	Feijão Manteigão	Domingos Martins, ES
IFES 71	Feijão Vagem Riscada	Jerônimo Monteiro, ES
IFES 72	Feijão Preto	Iúna, ES
IFES 73	Feijão Carioca	Iúna, ES
IFES 75	Feijão Terrinha	Iúna, ES
IFES 76	Feijão Branco	Alegre, ES
IFES 77	Feijão Preto	Comercial
IFES 78	Feijão Carioca	Comercial

**Table 5:** Quantitative descriptors used in the characterization of accessions from the Active Germplasm Bank of *Phaseolus vulgaris* L of IFES—Campus de Alegre, Brazil.

Descriptor	Method of Evaluation
PH—Plant height (cm)	Measured from the cotyledonary scar to the tip of the plant.
LL—Leaflet length (cm)	Measured on the terminal leaflet of the third trifoliate leaf from the base of the leaflet to its tip.
LW—Leaflet width (cm)	Measured on the terminal leaflet of the third trifoliate leaf from one edge to the other.
PW—Pod width (mm)	Measured from the largest immature but fully developed pods.
PL—Pod length (mm)	Measured from the largest immature but fully developed pods.
AL—Apex length (mm)	Measured from the end of the last locule.
NPP—Number of pods per plant (un)	Counted after flowering, between 40 and 50 days, with well-developed pods.
SL—Seed length (mm)	Measured parallel to the hilum.
SW—Seed width (mm)	Measured perpendicular to the hilum.
SH—Seed height (mm)	Measured from the hilum to the opposite side.
NSP—Number of seeds per pod (un)	Counted after harvest with the pod completely dry.
W100S—Weight of 100 seeds (g)	Measured after harvest, to the nearest first decimal place, with a moisture content of 12–14%.

**Table 6:** Qualitative descriptors used in the characterization of accessions from the Active Germplasm Bank of *Phaseolus vulgaris* L of Ifes—Campus de Alegre, ES, Brazil.

Descriptors	Classes
Growth habit	1 = determinate bushy; 2 = indeterminate bushy; 3 = indeterminate semi-vine or prostrate; 4 = indeterminate climbing.
Leaflet shape	1 = triangular; 2 = quadrangular; 3 = round.
Bracteole shape	1 = lanceolate; 2 = intermediate; 3 = ovate.
Ratio of bracteole length to calyx length	1 = shorter or equal; 2 = up to 1/3 longer; 3 = twice the length.
Color of standard	1 = white; 2 = green; 3 = lilac; 4 = white with lilac edges; 5 = white streaked with red; 6 = dark lilac with purple edges; 7 = dark lilac with purplish spots; 8 = crimson; 9 = purple; 10 = other.
Presence of anthocyanin in stem	0 = absent; 1 = present.
Pod apex orientation	1 = upwards (dorsal direction); 2 = straight; 3 = downwards (ventral direction).
Pod apex position	1 = marginal; 2 = non-marginal; 3 = other.
Seed shape	1 = round; 2 = oval; 3 = cuboid; 4 = kidney-shaped; 5 = truncated and elongated.
Seed brightness	1 = dull; 2 = medium; 3 = shiny.
Predominant seed coat color	1 = black; 2 = light to dark brown; 3 = reddish brown; 4 = grayish brown to greenish; 5 = yellow to yellow-green; 6 = light cream to dull yellow; 7 = pure white; 8 = whitish; 9 = white tinged with purple; 10 = chlorophyll green; 11 = olive green; 12 = red; 13 = pink; 14 = purple; 15 = other.
Secondary seed coat color	1 = black; 2 = light to dark brown; 3 = reddish brown; 4 = grayish brown to greenish; 5 = yellow to yellow-green; 6 = light cream to dull yellow; 7 = pure white; 8 = whitish; 9 = white tinged with purple; 10 = chlorophyll green; 11 = olive green; 12 = red; 13 = pink; 14 = purple; 15 = other.
Seed coat pattern	0 = absent; 1 = marbled; 2 = striped; 3 = rhomboid spotted; 4 = dotted; 5 = circular spotted; 6 = marginal color pattern; 7 = broad stripes; 8 = bicolor; 9 = bicolor spotted; 10 = hilum ring pattern; 11 = other.

**Table 7:** List of 23 ISSR primers used in molecular analysis to access diversity among 67 common bean accessions belonging to Active Germplasm Bank of IFES—Campus de Alegre, ES, Brazil.

Primer Code	Sequence	Annealing Temperature (°C)
A1	CACACACACACACACAAG	45
A3	GAGAGAGAGAGAGAGAT	45
A4	GAGAGAGAGAGAGAGAYC	49
A7	TCTCTCTCTCTCTCTCG	45
A11	AGAGAGAGAGAGAGAGYT	49
A12	AGAGAGAGAGAGAGAGYA	49
A17	GAGAGAGAGAGAGAGAYC	49
A18	ACACACACACACACACYG	49
A21	TGTGTGTGTGTGTGTGRA	49
A23	CTCCTCCTCCTCCTCCTC	49
A28	CTCTCTCTCTCTCTCTCTYA	49
P12	GATGATGATGATGATGAT	45
P13	GACAGACAGACAGACA	49
P14	GGATGGATGGATGGAT	45
P15	GGAGGAGGAGGAGGAGGA	45
P16	GGGTGGGGTGGGGTG	45
P17	GAGAGAGAGAGAGAGAA	49
P18	CTCTCTCTCTCTCTCTCTCTG	49
P19	CTCTCTCTCTCTCTCTCTCTT	49
P20	AGAGAGAGAGAGAGAGAGAGG	49
P21	AGAGAGAGAGAGAGAGAGAGC	49
P22	GACACGACACGACACGACAC	49
P23	ACTGACTGACTGACTG	49

## Data Availability

Data is contained within the article.
